# B2 SINE Copies Serve as a Transposable Boundary of DNA Methylation and Histone Modifications in the Mouse

**DOI:** 10.1093/molbev/msab033

**Published:** 2021-02-16

**Authors:** Tomoko Ichiyanagi, Hirokazu Katoh, Yoshinobu Mori, Keigo Hirafuku, Beverly Ann Boyboy, Masaki Kawase, Kenji Ichiyanagi

**Affiliations:** 1 Laboratory of Genome and Epigenome Dynamics, Department of Animal Sciences, Graduate School of Bioagricultural Sciences, Nagoya University, Nagoya 464-8601, Japan; 2 The Jikei University Hospital, Minato-ku, Tokyo 105-8471, Japan

**Keywords:** SINE, epigenetics, intra-specific difference, chromatin boundary, transposable elements

## Abstract

More than one million copies of short interspersed elements (SINEs), a class of retrotransposons, are present in the mammalian genomes, particularly within gene-rich genomic regions. Evidence has accumulated that ancient SINE sequences have acquired new binding sites for transcription factors (TFs) through multiple mutations following retrotransposition, and as a result have rewired the host regulatory network during the course of evolution. However, it remains unclear whether currently active SINEs contribute to the expansion of TF binding sites. To study the mobility, expression, and function of SINE copies, we first identified about 2,000 insertional polymorphisms of SINE B1 and B2 families within *Mus musculus*. Using a novel RNA sequencing method designated as melRNA-seq, we detected the expression of SINEs in male germ cells at both the subfamily and genomic copy levels: the vast majority of B1 RNAs originated from evolutionarily young subfamilies, whereas B2 RNAs originated from both young and old subfamilies. DNA methylation and chromatin immunoprecipitation-sequencing (ChIP-seq) analyses in liver revealed that polymorphic B2 insertions served as a boundary element inhibiting the expansion of DNA hypomethylated and histone hyperacetylated regions, and decreased the expression of neighboring genes. Moreover, genomic B2 copies were enriched at the boundary of various histone modifications, and chromatin insulator protein, CCCTC-binding factor, a well-known chromatin boundary protein, bound to >100 polymorphic and >10,000 non-polymorphic B2 insertions. These results suggest that the currently active B2 copies are mobile boundary elements that can modulate chromatin modifications and gene expression, and are likely involved in epigenomic and phenotypic diversification of the mouse species.

## Introduction

Short interspersed elements (SINEs) are a class of transposable elements present in a wide variety of organisms (Vassetzky and Kramerov 2013) that originated from RNA polymerase III (Pol III)-transcribed genes such as tRNAs, 7SL RNA, and 5S rRNA (Ullu and Tschudi 1984; Daniels and Deininger 1985; Sakamoto and Okada 1985; Kapitonov and Jurka 2003), thus, SINE transcription depends on Pol III. Transposition involves reverse transcription of SINE RNAs and integration of the resultant cDNAs into a genomic DNA site, a process termed retrotransposition (Ichiyanagi 2013). While SINEs do not encode a protein, long interspersed elements (LINEs) present in the same genome provide a reverse transcriptase activity for SINE retrotransposition (Kajikawa and Okada 2002; Dewannieux et al. 2003; Dewannieux and Heidmann 2005). Due to the absence of protein-coding genes within these elements, SINE transcription alone does not cause a mutation by retrotransposition; rather, it has been proposed that SINE RNAs have functions regulating gene transcription and translation (Chu et al. 1998; Allen et al. 2004; Espinoza et al. 2004; Mariner et al. 2008). Moreover, it has been shown that a variety of ancient SINE families—LF-SINE, AmnSINE1, MIR, B3, B4, SINEC, and Mar1—have provided *cis* regulatory sequences during the course of mammalian evolution (Nishihara 2019).

SINEs exhibit tissue-specific expression patterns, implying they undergo epigenetic regulation. However, the related underlying mechanisms remain conjectural in that DNA methylation at CpG sites was proposed to play a role in repression of Pol III transcription (Besser et al. 1990; Englander et al. 1993; Liu and Schmid 1993), whereas later studies with cultured cells suggest that histone H3 lysine 9 (H3K9) methylation is more important for repression (Kondo and Issa 2003; Varshney et al. 2015).

The murine genome contains the SINE families of B1, B2, B3, B4, BC1, ID, MIR and AmnSINE1, encompassing a total of ∼1.4 million copies that occupy ∼7% of the genome (Waterston et al. 2002). The 7SL RNA-derived B1 family contains retrotranspositionally active copies (Dewannieux and Heidmann 2005). We previously reported that the B1 family is most highly expressed in testes, and while some copies show very low levels of DNA methylation in spermatogenic cells, they are heavily methylated in somatic cells (Ichiyanagi et al. 2011). The tRNA-derived B2 family also contains retrotranspositionally active copies (Dewannieux and Heidmann 2005). It is of interest that a murine B2 copy has been shown to constitute a boundary between chromatin domains enriched with H3K9 trimethylation (H3K9me3) and H3K9 dimethylation (H3K9me2), respectively (Lunyak et al. 2007). Accordingly, chromatin immunoprecipitation with sequencing (ChIP-seq) studies revealed that in the mouse strain C57BL6/J (B6), approximately 39,000 B2 copies (about 32% of all genomic B2 copies) carry binding sites for a chromatin insulator protein, CCCTC-binding factor (CTCF) or its binding competitor, activity-dependent neuroprotective protein (ADNP) (Bourque et al. 2008; Schmidt et al. 2012; Thybert et al. 2018; Kaaij et al. 2019). The B2-related ancient SINEs, B3 and B4, also provide a larger number of CTCF binding sites (Schmidt et al. 2012).

In this study, we delineated recent SINE retrotransposition and its impacts on the host epigenome and transcriptome. First, the recent retrotranspositional activity of B1 and B2 was investigated by identification of insertional polymorphisms between two mouse strains. Second, the expression profiles of SINEs were analyzed based on Northern blotting and a deep sequencing method specifically developed for this study. Finally, DNA methylation and ChIP-seq analyses revealed that a recently retrotransposed B2 copy formed a boundary in terms of DNA methylation and histone acetylation, and down-regulated its neighboring gene. Further analysis identified another instance in which a polymorphic B2 insertion created a chromatin boundary and down-regulated the neighboring gene. In addition, a total of > 200 insertionally polymorphic B2 copies retained their CTCF- or ADNP-binding ability. We explore the possibility that B2 retrotransposition has been involved in divergence of the host epigenome and gene expression pattern during the course of evolution.

## Results

### Genomic Analysis of SINE Copies

**Fig. 1.**
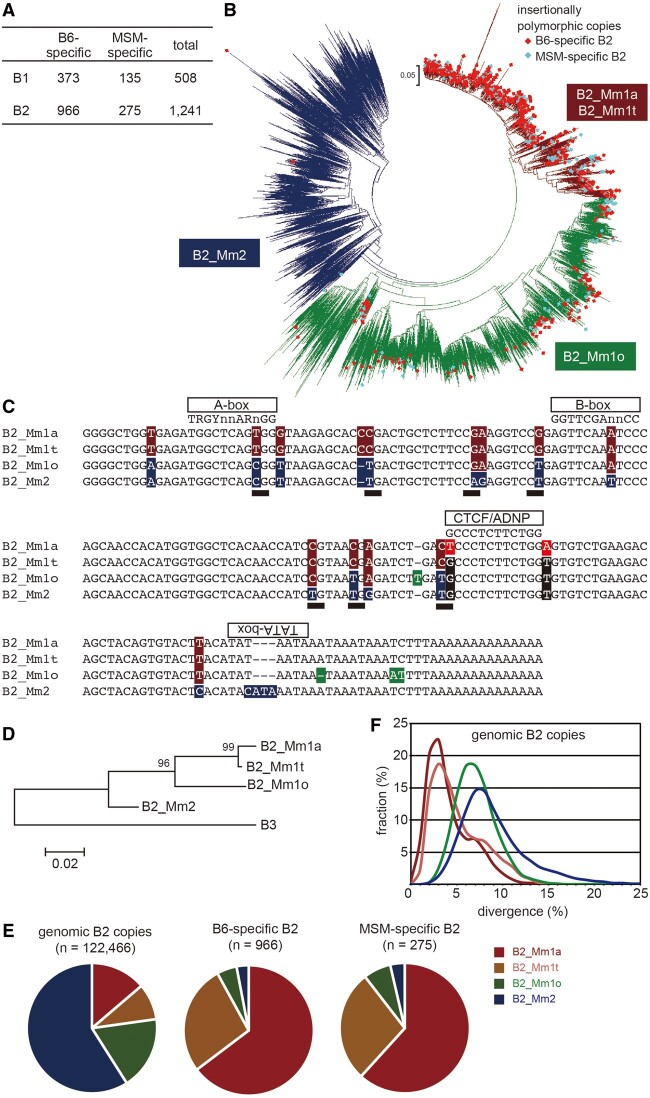
B2-generated indels between B6 and MSM.(*A*)Statistics of the indels identified in this study. (*B*)A neighbor-joining tree of 1,241 B2-derived indels and 6,000 B2 copies randomly selected from the reference genome sequence. B6- and MSM-specific copies are indicated in red and light blue diamonds, respectively. (*C*)The alignment of the consensus sequences of B2 subfamilies. The sequences for B2_Mm1a, B2_Mm1t, and B2_Mm2 were obtained from Repbase; B2_Mm1o consensus sequence was generated in the current study. A-, B-, and TATA-box as well as the CTCF/ADNP binding motif are indicated above the sequences. Nucleotides that are different between the subfamilies are highlighted. CpG sites found in at least one consensus sequence are indicated by filled rectangle. (*D*)Phylogenetic tree of the B2 subfamilies. The values on the clades indicate bootstrap values. Scale bar shows divergence rate (substitutions per site). (*E*)Pie chart representations of the numbers for genomic (left), B6-specific (middle), and MSM-specific (right) B2 copies categorized by subfamily. (*F*)Divergence of genomic B2 copies from the respective consensus sequences. Color codes for panels (*E*) and (*F*) are shown on the lower right.

To study recent SINE retrotranspositional activity, we identified insertional polymorphisms between two laboratory mouse strains, C57BL6/J (B6) and MSM/Ms (MSM), derived from different subspecies that diverged approximately one million years ago. To identify insertions and deletions (indels) differing between these strains, we blasted the shotgun sequencing reads of the MSM genome (Takada et al. 2013) against the B6 genome (i.e. mouse reference genome) using the minimum gap penalty (see Materials and Methods section). This yielded a total of 4,578 indels ranging from 120 to 300 bp in length. These indel sequences were then analyzed by RepeatMasker, which revealed that 508 were generated by B1 retrotransposition and 1,241 by B2 retrotransposition (fig. 1*A*;supplementary table S1, Supplementary Material online). In the reference genome, the copy number of B2 (about 120,000) is lower than that of B1 (about 420,000). Therefore, the higher number of insertional polymorphisms within B2 compared to B1, together with the higher *in vitro* retrotransposition activities observed for B2 (Dewannieux and Heidmann 2005), suggests that B2 has been more active than B1 since their divergence occurred. The number of MSM-specific insertions were three times lower than B6-specific insertions. This suggests a lower retrotransposition rate in the MSM strain, although it is also possible that an assortment bias exists: for example, about 200-bp insertions could be more preferentially identified in the assembled reference genome than those in shotgun sequencing reads.

Subfamily analysis of the insertionally polymorphic B1 copies (373 B6-specific and 135 MSM-specific) revealed that the vast majority belonged to B1_Mm, B1_Mus1, or B1_Mus2 (supplementary fig. S1, Supplementary Material online). These copies showed very limited divergence from the respective consensus sequences. In contrast, very few copies were polymorphic in the case of the older subfamilies (B1_Mur1, B1_Mur2, B1_Mur3, B1_Mur4, B1F, B1F1, and B1F2).

Similarly, phylogenetic analysis of B6- and MSM-specific B2 copies and 6,000 copies randomly selected from the reference sequence revealed that most of the polymorphic copies were clustered with those of B2_Mm1a and B2_Mm1t in the phylogenetic tree (fig. 1*B*; brown). In contrast, very few polymorphic copies were located in the B2_Mm2 clade (fig. 1*B*; blue). The remaining polymorphic copies were positioned between the B2_Mm1 and B2_Mm2 clades (fig. 1*B*; green); we hypothesized that this clade represents an unidentified subfamily. Thus, we constructed the consensus sequence using copies of this clade, disclosing that it carries both B2_Mm1-specific and B2_Mm2-specific nucleotides (fig. 1*C*). These results, together with the structure of the phylogenetic tree (fig. 1*D*) and numbers of polymorphic sites (fig.1*E*) suggest that this subfamily is evolutionally older than the B2_Mm1a and B2_Mm1t subfamilies. We designated this unidentified subfamily as B2_Mm1o (i.e.older). Using RepeatMasker, we reassigned all B2 copies in the reference genome sequence, and the new annotation criteria yielded about 17,000 (B2_Mm1a), 11,000 (B2_Mm1t), 22,000 (B2_Mm1o), and 72,000 (B2_Mm2) copies (supplementary table S2, Supplementary Material online). Nucleotide divergences of genomic copies from the respective consensus sequences (fig. 1*F*) were consistent with the following postulated evolutionary history: B2_Mm2 as oldest, B2_Mm1o as intermediate, B2_Mm1a and B2_Mm1t as the youngest. In comparison to B2_Mm1a and B2_Mm1t, much fewer B2_Mm1o copies were insertionally polymorphic (fig. 1*E*), although the genomic copy number of B2_Mm1o was higher than B2_Mm1a and B2_Mm1t.

### B2 RNA Expression in Testis

**Fig. 2.**
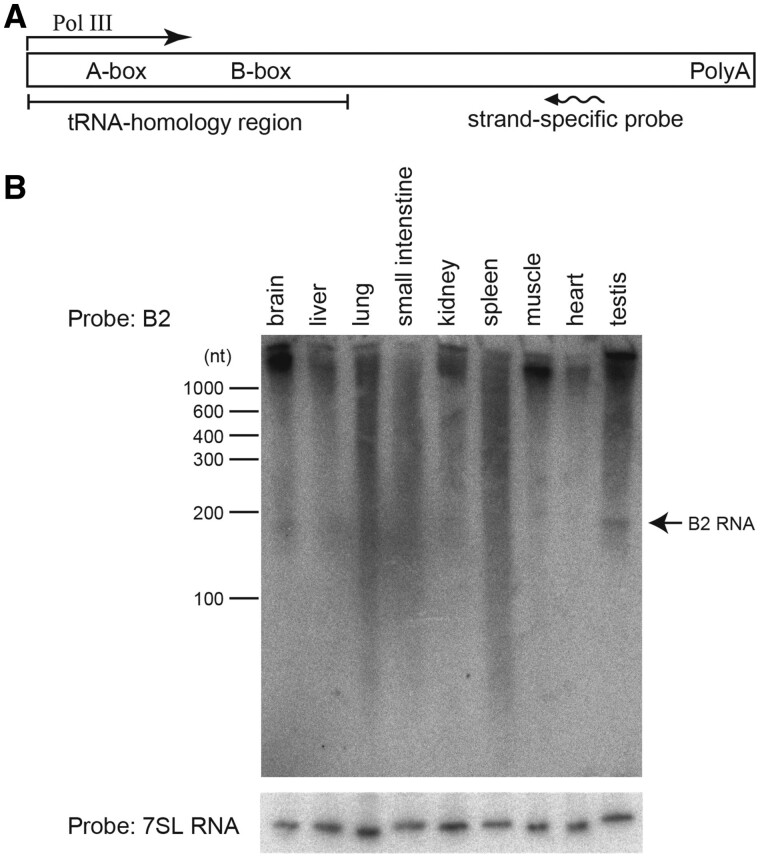
Expression analysis of B2 within tissues. (*A*)Position of the oligonucleotide probe used in panel *B*. (*B*)Northern blot results for somatic tissues and testis. The gel mobility of RNA markers (100–1,000 nt) is shown on the left, and the mobility of B2 RNA (∼190 nt) is indicated on the right; 7SL RNA (bottom) was used as an internal control.

PCR or conventional RNA deep sequencing (i.e. mRNA-seq) cannot give precise amounts of SINE RNAs transcribed by their own promotors, because many SINE sequences reside in Pol II transcripts such as mRNAs. Using Northern blot analysis, we previously showed that B1 is expressed specifically in the testes (Ichiyanagi et al. 2011). Here, we investigated the B2 expression profile in adult mouse tissues by Northern blotting (fig. 2), which can detect B2 RNAs of ∼190 nucleotides. The analysis revealed that, similarly to B1, B2 was expressed in testes, whereas it was very weakly expressed in somatic tissues.

### DNA Methylation Profile of Individual B2 Loci in Somatic and Spermatogenic Cells

DNA methylation is an important epigenetic modification for regulating the activity of Pol II promoters, although its role in Pol III regulation remains conjectural. Therefore, the DNA methylation levels of B2 copies were analyzed for spermatogenic cells (spermatogonia and spermatozoa) and in the liver as a somatic control in which B2 expression was scarcely detected.

**Fig. 3.**
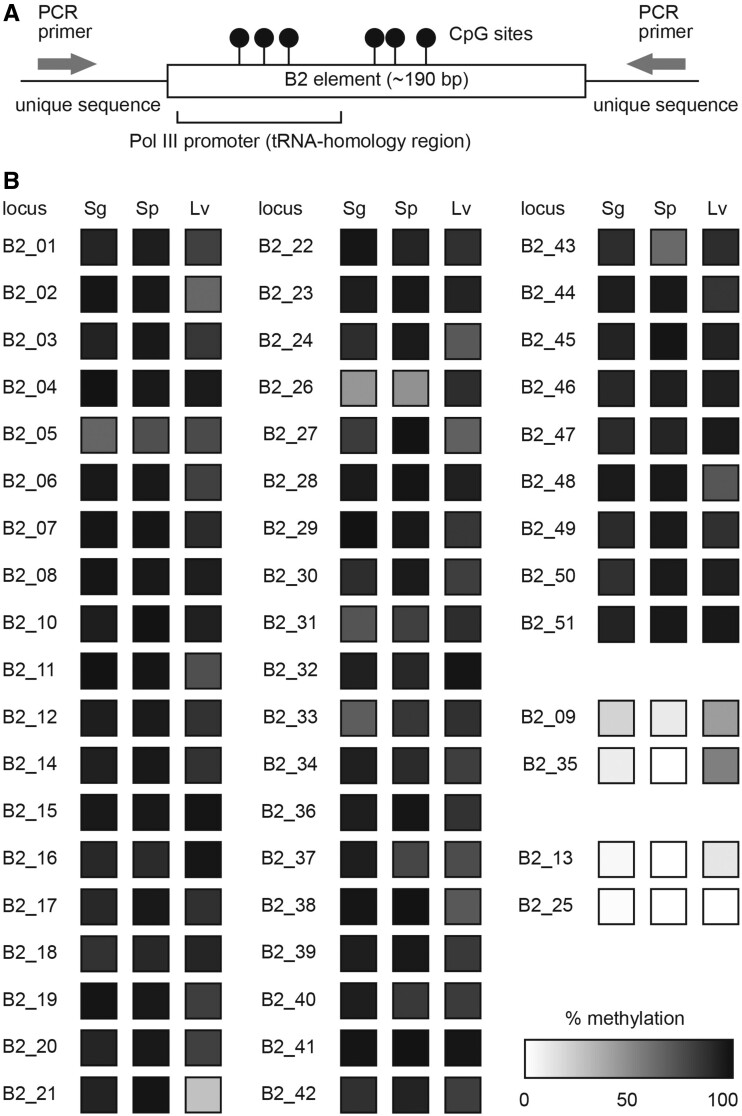
DNA methylation analysis of B2 copies using the bisulfite PCR method.(*A*)Schematic representation; lollipops indicate 4–8 CpG sites in the B2 sequences analyzed; PCR primers were designed for both flanking regions. (*B*)DNA methylation levels of 51 genomic B2 copies in spermatogonia (Sg), spermatozoa (Sp), and liver (Lv) are shown in grayscale.

For bisulfite-PCR sequencing analysis, we randomly selected 51 B2 copies from both polymorphic and non-polymorphic copies that had at least 4 CpG sites (supplementary table S3, Supplementary Material online), and the results revealed that most copies (47 of 51; 92%) were heavily methylated in both spermatogenic and liver cells (fig. 3). Only two copies (B2_09 and B2_35) showed a difference in methylation with heavier methylation in liver vs. spermatogenic cells. The other copies (B2_13 and B2_25) were primarily unmethylated in all cells analyzed. Thus, overall methylation levels of B2 copies did not differ significantly between germ and somatic cells.

### melRNA-Seq Analysis of B2 Expression in Germ Cells

**Fig. 4.**
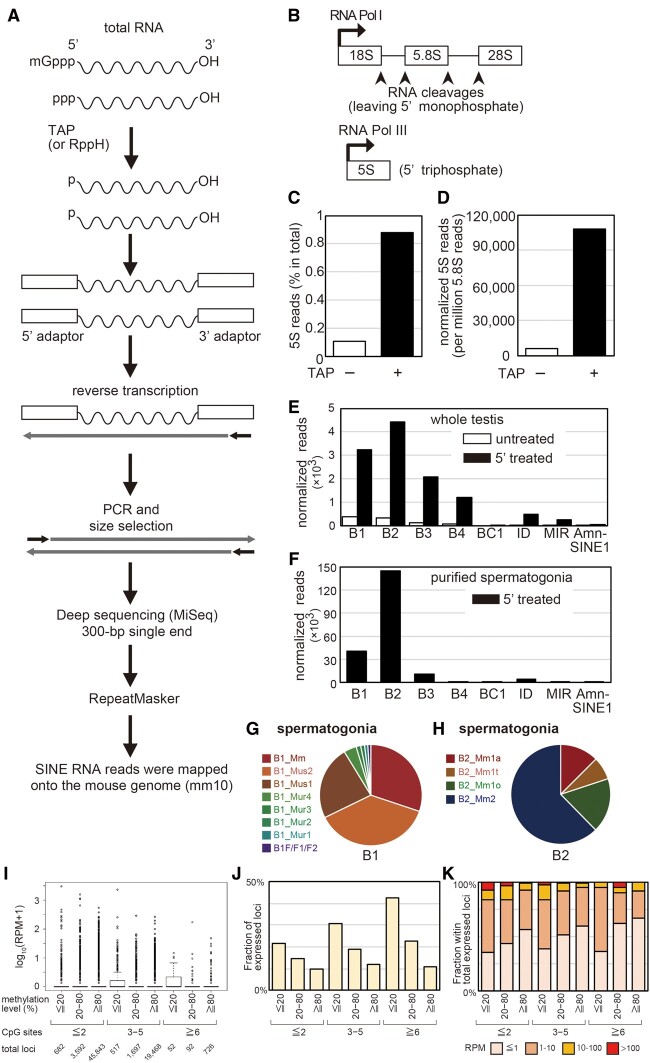
melRNA-seq analysis.(*A*)Schematic representation of melRNA-seq methodology. (*B*)Schematic representation of biosynthesis of 5.8S (top) and 5S (bottom) rRNAs. (*C*)The fraction (%) of 5S RNA reads in TAP-untreated (−) and -treated (+) melRNA-seq libraries prepared from testis RNA. (*D*)Fraction of 5S RNA reads normalized with 5.8S RNA reads in the testis. (*E*)5.8S RNA-normalized read counts of the SINE families in the testis library. White, TAP-untreated RNA. Black, TAP-treated RNA. F. 5.8S RNA-normalized read counts of the SINE families in the 5’-treated spermatogonia library. (*G* and *H*)Pie chart representations of read counts of B1 and B2 subfamilies, respectively, in spermatogonia. (*I*)Boxplots for expression levels of B2 loci categorized by the number of CpG sites and the level of methylation in spermatogonia. (*J*)Fraction of B2 loci that were expressed (at least one sequencing reads were mapped). The loci are categorized by the number of CpG and the level of methylation in spermatogonia. (*K*)Fraction of expressed loci giving an RPM value of 1 or less, 1–10, 10–100, and over 100. The loci are categorized by the number of CpG and the level of methylation in spermatogonia.

Since Northern blotting does not discriminate subfamilies, we developed a new sequencing method to analyze SINE transcripts (fig. 4*A*, see Material and Methods for details). Briefly, we first converted the cap and triphosphate at the 5’ end of the RNA to a 5’ monophosphate by treating total testis RNA with tobacco alkaline phosphatase (TAP). Then, RNA adaptors were ligated to both ends of the RNA for cDNA synthesis and PCR amplification. After selection based on insert size, the libraries were sequenced on a MiSeq via 300-bp single-end sequencing. Sequencing reads containing the 3’ adaptor sequence should represent the entire RNA molecule from the 5’ to 3’ ends. These reads were retained and analyzed by RepeatMasker.

First, we evaluated the usability of the developed sequencing method based on sequence reads of the 5.8S (158 nt) and 5S (121 nt) rRNAs generated via different pathways: 5.8S rRNA carries a ligatable 5’ monophosphate generated by enzymatic cleavages of the 45S rRNA precursor, whereas Pol III-transcribed 5S rRNA contains a 5’ triphosphate end (fig. 4*B*). Regardless of enzymatic processing, many reads were derived from 5.8S rRNA as expected. In contrast, when total RNAs were not treated with TAP, very few reads (0.1% of total) were derived from 5S rRNA (fig. 4*C*). TAP treatment increased the recovery of 5S rRNA sequences by 18-fold when 5.8S rRNA reads were used as an internal control (fig. 4*D*). These results validated the accuracy of this newly developed method, in that intact RNA transcripts of about 100–300 nt in length, including those generated by RNA Pol III, were captured in sequencing libraries and analyzed at the nucleotide level. We designated this method as melRNA-seq (sequencing of medium length RNAs). RepeatMasker analysis of the sequencing reads of TAP-treated RNAs detected substantial amounts of B1 and B2 reads, and smaller numbers of B3, B4, BC1, ID, MIR, and AmnSINE1 (fig. 4*E*). Very few SINE reads were detected in the library from untreated RNAs, indicating that these sequence reads were derived from Pol III transcripts rather than from degradation products of long transcripts.

Since testis contains both somatic and germ cells, we constructed melRNA-seq libraries from FACS-purified spermatogonia at postnatal Day 7 to see if SINEs are expressed in germ cells. The results (fig. 4*F*) again showed high expression of B1 and B2: about 0.5% and 2.2% of sequence reads were assigned as B1 and B2, respectively. The expression levels normalized by 5.8S rRNA reads were higher in spermatogonia than those in testis by about 10-fold (B1) and 40-fold (B2) (compare fig. 4*E* and *F*), suggesting that the expression of B1 and B2 are much higher in germ cells than testicular somatic cells. Most (91%) of B1 RNAs were derived from the youngest subfamilies, B1_Mm, B1_Mus2, and B1_Mus1 (fig. 4*G*). Thus, the level of expression of each B1 subfamily is proportional to the number of insertionally polymorphic copies. In contrast, the B2 RNA profile indicated that the youngest subfamilies, B2_Mm1a and B2_Mm1t, contributed to only 20% of the total B2 RNAs (fig. 4*H*), and the majority were derived from the older subfamilies B2_Mm2 (62%) and B2_Mm1o (18%). When the B2-derived reads were mapped onto the reference mouse genome, about 40% of them were mapped uniquely to a single locus with no mismatch. Two biological replicates showed good accordance (Pearson’s*R* = 0.997 at subfamily level and R = 0.93 at locus level; Supplementary Fig. S2), and identified a total of 8,088 expressed loci with a maximal RPM (reads per million 5.8S rRNA reads) of about 3,000. The expression levels largely followed the power law (supplementary fig. S3, Supplementary Material online), and varied by three orders of magnitude. Thus, more than a half of the loci (4,352 loci) showed RPM values of <1, while only 5 loci showed RPM of >1,000.

Using published DNA methylome data for spermatogonia at postnatal Day 7 (Kubo et al. 2015), methylation levels of individual loci were analyzed. Since the number of CpG sites in a copy varied largely, we categorized B2 copies based on the CpG number as well as the methylation level. This revealed that, for B2 copies with ≧3 CpG sites, the expression levels were higher in loci with lower methylation (fig. 4*I*). Larger fractions of loci were expressed (at least one sequence reads in either replicate were mapped) in the groups with lower methylation levels (fig. 4*J*). Moreover, a tendency was observed where loci showing a lower methylation level gave higher expression (fig. 4*K*). But, we also note that the effect of DNA methylation became less in CpG-poor B2 loci (compare the three categories by CpG number in fig. 4*J*). Therefore, in spermatogonia, the level of CpG methylation was one of determinants of the expression levels for CpG-rich, evolutionally young copies, but if mutations at CpG sites were accumulated, DNA methylation had less impact on the expression.

### B2 Copies Formed Boundaries of DNA Methylation and Histone Modification Domains

**Fig. 5.**
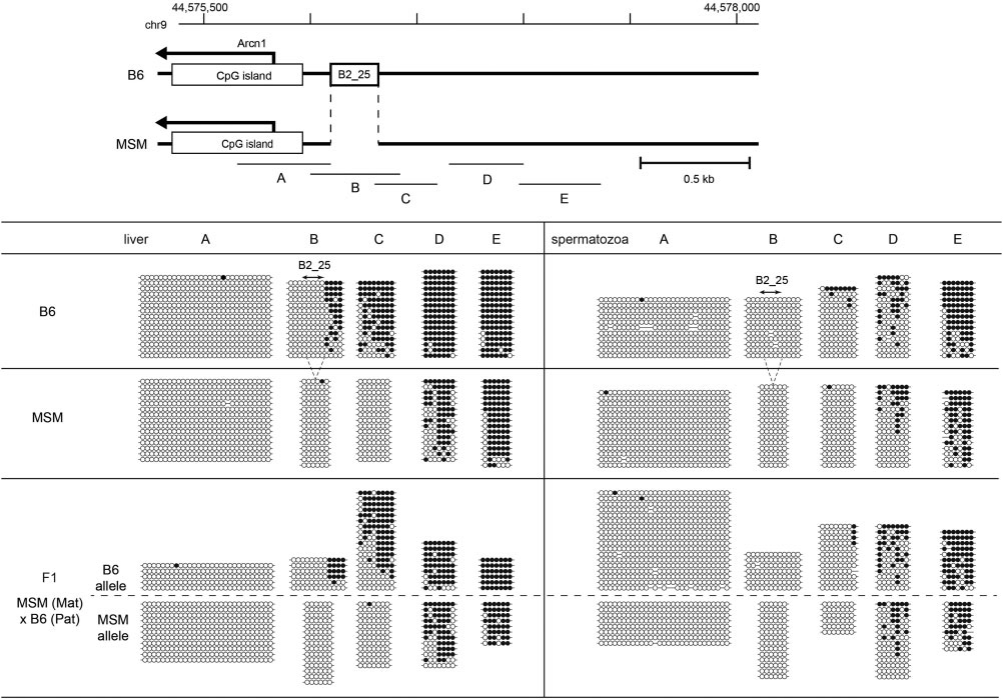
DNA methylation states surrounding B2_25 in B6, MSM, and F1 mice.Methylation status of the regions A–E in liver and spermatozoa are shown; open and closed circles represent unmethylated and methylated CpG sites, respectively. Each horizontal line represents a single PCR clone. The position of the B2_25 insertion is indicated.

During the course of our study on methylation of individual B2 loci shown in fig. 3, we noted that B2_25—a copy of B2_Mm1a that is unmethylated in all cell types (fig. 3 and supplementary fig. S4, Supplementary Material online)—was located between regions that were unmethylated and heavily methylated in liver (fig. 5). This locus was located 200 bp upstream of the transcription start site (TSS) of the *Arcn1* gene. This B2 copy and the region around the TSS were unmethylated; however, the four CpG sites adjacent to the B2 sequence (fig. 5, Region B) as well as those in regions further upstream (fig. 5, Regions C − E) were heavily methylated. Therefore, this B2 copy resides at a boundary separating hypomethylated and hypermethylated domains. Such a boundary effect was also observed in the brain, spleen, lung, muscle, small intestine, and kidney (supplementary fig. S4, Supplementary Material online). In sperm, the hypomethylated region around the TSS became wider as seen with many CpG islands (Molaro et al. 2011), thus the methylation boundary translocated to a further upstream region (fig. 5, Regions D and E). These results suggest the possibility that in somatic tissues a hypomethylated region is formed across the TSS, but does not extend beyond B2_25. To test this, we analyzed DNA methylation levels in the liver of MSM mice where this B2 copy is absent, and revealed that the hypomethylated region was extended to the upstream region (fig. 5). The methylation difference between the strains was also observed as an allelic difference in F1 hybrids, indicating the *cis* effect of the B2_25 insertion as a DNA methylation boundary.

**Fig. 6.**
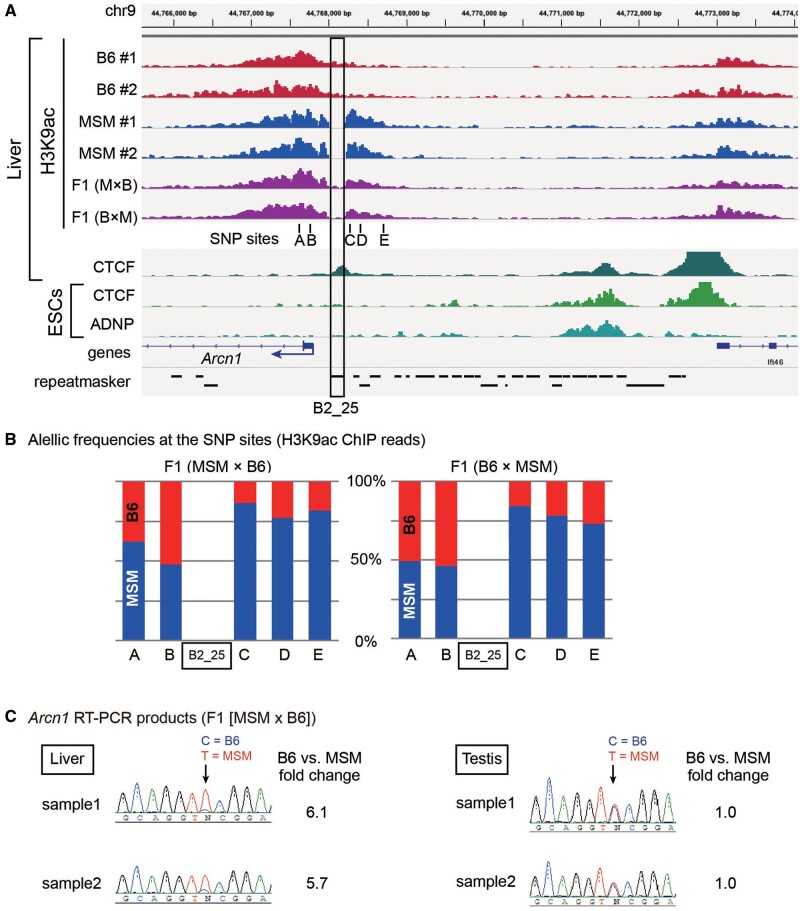
Chromatin boundary formed at B2_25.(*A*)ChIP-seq data of H3K9 acetylation (H3K9ac) for B6 (two individuals, red), MSM (two individuals, blue), and F1 hybrids (generated by reciprocal crosses, purple). (*B*)Allelic frequencies in the ChIP-seq reads obtained from the F1 hybrids are shown at known SNP positions (A–E) indicated in panel *A*. (*C*)Electrophoregrams from Sanger sequencing analysis of RT-PCR products for *Arcn1* in liver and testis from F1 hybrids.

Because DNA methylation likely reflected histone modification status, we carried out ChIP-seq analysis for histone H3 lysine 9 acetylation (H3K9ac) using livers from B6, MSM, and their hybrids (fig. 6*A*). We detected a ChIP-seq peak in the regions surrounding the TSS of *Arcn1* in both strains. Importantly, the hyperacetylated region ended at the site of B2_25 insertion in B6, whereas it was extended upstream in MSM mice. ChIP-seq results of the reciprocal F1 hybrids showed an allelic difference in the region beyond B2_25, where the MSM allele was hyperacetylated (fig. 6*B*), again indicating the *cis* effect of B2_25 insertion.

The H3K9ac modification is involved in the transcriptional activation, and the length of the modified region has been proposed to be correlated to the robustness of chromatin states (Dodd et al. 2007). Consistent with the difference in the size of the H3K9ac peak, transcription of the *Arcn1* gene in the liver was about 6-fold higher in the MSM (B2-free) allele vs. the B6 (B2-containing) allele within the F1 hybrid mice (fig. 6*C*). These results suggest that, by inhibiting the expansion of active chromatin, this B2 insertion is involved in intra-species divergence of the expression of *Arcn1*. Such boundary function may suggest binding of CTCF, a well-known chromatin boundary protein. Indeed, published CTCF ChIP-seq data for liver disclosed the CTCF binding at the site of this B2 copy (fig. 6*A*).

**Fig. 7.**
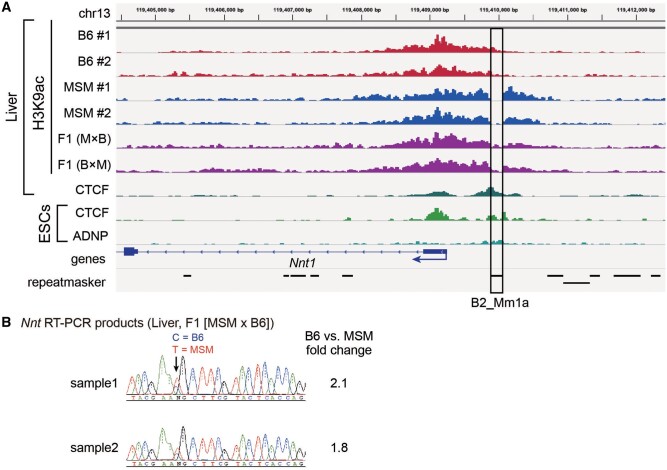
Chromatin boundary formed at the B2 copy upstream of *Nnt*.(*A*)ChIP-seq data of H3K9 acetylation (H3K9ac) for B6 (two individuals, red), MSM (two individuals, blue), and F1 hybrids (generated by reciprocal crosses, purple). (*B*)Electrophoregrams from Sanger sequencing analysis of RT-PCR products for *Nnt* in liver of F1 hybrids.

To extend our finding, we also searched for insertionally polymorphic B2 copies residing at the ChIP-seq peak boundary, and identified a B2_Mm1a copy in the upstream region of the TSS of the *Nnt* gene (fig. 7*A*). Consistent with the size difference in the H3K9ac peak, the MSM (B2-free) allele showed ∼2-fold higher expression than the B6 (B2-containing) allele within F1 hybrids (fig. 7*B*). In this instance, again we detected a CTCF peak in liver (fig. 7*A*). Moreover, based on published ChIP-seq data for CTCF and ADNP, a binding competitor of CTCF, in embryonic stem cells (ESCs) (Kaaij et al. 2019), this copy was occupied by both proteins.

**Fig. 8.**
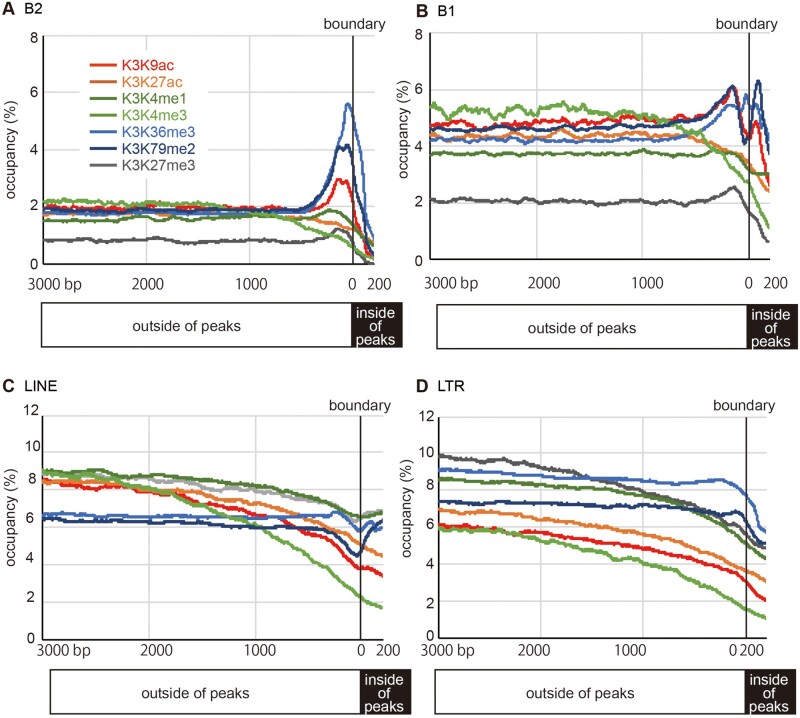
Enrichment of genomic B1 and B2 copies at Chromatin boundaries.Genomic occupancies of B2 (*A*), B1 (*B*), LINE (*C*), and LTR elements (*D*) are shown in the indicated positions with respect to the boundaries of ChIP-seq peaks of various histone modifications. The color codes for histone modifications are shown in panel *A*. The numbers of ChIP-seq peaks are 29,230 (H3K9ac), 38,492 (H3K27ac), 77,192 (H3K4me1), 16,888 (H3K4me3), 88,353 (H3K36me3), 68,593 (H3K79me2), and 33,402 (H3K27me3).

**Fig. 9.**
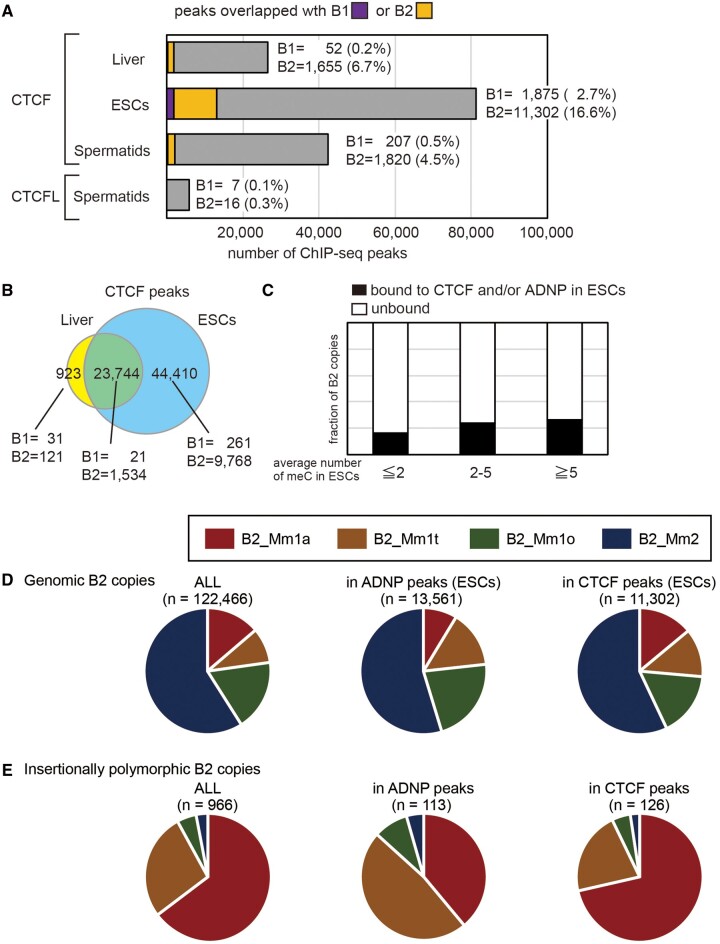
Genomic B2 copies form CTCF, CTCFL, and ADNP binding sites.(*A*)The numbers ChIP-seq peaks for CTCF, CTCFL, and ADNP in liver, ESCs, and round spermatids. Peak summits within genomic B1 and B2 copies are colored purple and orange, respectively. The exact numbers and proportions (%) are shown on the right. (*B*)Venn diagram of CTCF peaks identified in liver and ESCs. The numbers of B1 and B2 copies overlapped with liver-specific, shared, and ESC-specific copies are shown on the bottom. (*C*)Statistics of DNA methylation levels for B2 copies in terms of DNA methylation and CTCF binding. Genomic B2 copies are categorized according to the number of methylated CpG sites in ESCs. (*D*and *E*)Pie chart representation of B2 subfamilies in total, CTCF-bound, ADNP-bound genomic (*D*), and polymorphic copies (*E*).

To expand these findings, we analyzed the ENCODE data (Iwasaki et al. 2020), revealing enrichment of B2 copies in regions neighboring ChIP-seq peaks of H3K9ac, H3K36me3, and H3K79me2 (fig. 8*A*). Weak enrichment was seen around H3K4me1 and H3K27me3 peak boundaries, whereas B2 was not enriched in H3K27ac and H3K4me3 boundaries. B1 was slightly enriched in peak boundaries of H3K9ac, H3K36me3, and H3K79me2 (fig. 8*B*), whereas LINEs and LTR elements were not enriched in peak boundaries (fig. 8*C* and *D*). These results suggest that, regardless of insertional polymorphism, B2 copies tend to comprise boundaries of histone modifications genome-wide. Similarly, analysis of published CTCF ChIP-seq data revealed that many genomic copies of all B2 subfamilies comprised CTCF binding sites in liver, ESCs, and spermatids (haploid male germ cells) (fig. 9*A*), which is consistent with previous reports (Bourque et al. 2008; Schmidt et al. 2012; Thybert et al. 2018; Kaaij et al. 2019). Especially, CTCF bound to many genomic sites in ESCs (fig. 9*B*), and B2 copies accounted for 16% of these CTCF binding sites (fig. 9*A*). The binding capacity was not related to the number of methylated cytosines in the respective B2 copies (fig. 9*C*). In addition, over 13,000 B2 copies from the all subfamilies bound to ADNP in ESCs (fig. 9*D*). On the other hand, CTCFL (also called BROIS), a germline-specific CTCF-like protein with a similar binding specificity, merely bound to B2 copies in spermatids (fig. 9*A*). It should be emphasized that more than one tenth of recently retrotransposed B2 copies, most of which are B2_Mm1a or B2_Mm1t, have resulted in the generation of new CTCF/ADNP-binding sites during recent mouse evolution (fig. 9*E*). Therefore, it is likely that, rather than the CTCF/ADNP-binding activity being acquired by undergoing sequence changes, this activity is built-in the original B2 copies of retrotranspositional expansion. It is of note that the consensus sequences of B2 subfamilies harbor the CTCF binding motif (fig. 1*C*). However, we noticed that most of boundary-associated B2 were not bound to CTCF (supplementary fig. S5*A*, Supplementary Material online). Therefore, whereas CTCF bound to the *Arcn1*- and *Nnt*-proximal B2 copies, CTCF binding is not a general requirement for the B2 function to serve as a boundary of histone-modified regions. The fractions of subfamilies were largely proportional to their genomic copy numbers (supplementary fig. S5BC, Supplementary Material online).

On the other hand, B1 contributed a very minor fraction of CTCF binding sites (fig. 9*A*), and no insertionally polymorphic B1 copies bound to CTCF (supplementary fig. S1, Supplementary Material online).

## Discussion

It is known that a number of retrotransposon insertions are polymorphic between inbred mouse strains, such as B6, A/J, DBA2/J, 129S1/SvImJ, and 129X1/SvJ(Akagi et al. 2010), which are implicated in intra-species differences in gene expression and heterochromatin formation (Rebollo et al. 2011; Li et al. 2012; Akagi et al. 2013; Inoue et al. 2017). Here, we identified a total of ∼1,700 B1 and B2 insertions that are polymorphic between the B6 and MSM strains, which are of different subspecies origin (fig. 1). We note that the insertional difference between two inbred mouse lines of different subspecies origin does not necessarily indicate that the element is polymorphic between the two subspecies. There could be insertional polymorphism even within a subspecies, and inbreeding could have fixed one of the states in the given mouse line. Even if so, it is evident that these polymorphic SINE insertions represent recent retrotransposition events and give us a clue to explore the impact of their retrotransposition on the gene regulation.

Most insertionally polymorphic B1 copies belong to young subfamilies as expected, including B1_Mm, B1_Mus1, and B1_Mus2. Likewise, insertionally polymorphic B2 copies comprise the B2_Mm1a and B2_Mm1t subfamilies, whereas the B2_Mm2 subfamily has almost become extinct (fig. 1). During phylogenetic analysis, we identified a cluster distinct from B2_Mm1a and B2_Mm1t, which is designated here as B2_Mm1o. Many copies of the B2_Mm1o subfamily exhibit insertional polymorphism between the strains, but much less than those of B2_Mm1a and B2_Mm1t. A notable possibility is that the polymorphic B2_Mm1o copies could be due to ancestral polymorphism as they may have retrotransposed prior to divergence of the two subspecies, followed by incomplete lineage sorting.In any event, retrotransposition activity of the B2_Mm1o subfamily may have been severely decreased or already lost, while still retaining transcription capacity.

SINE expression levels cannot be precisely determined via conventional RT-qPCR and mRNA-seq analyses, because many SINE copies reside within mRNAs, particularly in the 3’ UTRs. Northern blotting can discriminate *bona fide* SINE RNAs and long RNAs carrying a SINE sequence based on differences in length. Therefore, we employed Northern blotting here and in a previous study (fig. 2; Ichiyanagi et al. 2011) to detect both types of RNAs hybridizing to B1- or B2-specific probes, and revealed that verified SINE RNAs are produced specifically within the testes in males; however, expression at the subfamily level could not be determined. A major technical advance in this study is the development of melRNA-seq for massive sequencing of whole length SINE RNAs, thereby precisely determining the expression levels of SINE subfamilies and genomic copies (fig. 4). SINE RNAs transcribed by RNA Pol III should have a triphosphate or a cap at the 5’ ends, which can be enzymatically removed for RNA ligation to prepare sequencing libraries. After validating the method principle by examining the read counts of 5.8S and 5S rRNAs, we detected SINE RNA expression in spermatogonia, which are developing germ cells present in testis. In the case of B1, the vast majority originated from copies of retrotranspositionally active subfamilies, suggesting that RNA abundance is a major determinant of retrotransposition activity. In the case of B2, however, RNA abundance is similar to the abundance of the genomic copies of each subfamily, and is not related to recent retrotransposition activity. Thus, other factors, such as the affinity of L1-encoded reverse transcriptase (Dewannieux and Heidmann 2005), may be involved in the efficiency of retrotransposition. The melRNA-seq analysis established here offers a comprehensive method to analyze cell type-specific expression of a variety of medium length non-coding RNAs, including not only SINEs but also tRNA species, snRNAs, and snoRNAs when libraries are prepared based on appropriate insert lengths.

The effect of DNA methylation on Pol III transcription seems to differ between individual genes. Enzymatic methylation of CpG sites in the DNA template has been demonstrated to inhibit the Pol III transcription of a tRNA gene when injected into *Xenopus* oocytes (Besser et al. 1990) and a human *Alu* SINE copy when added to nuclear extracts (Englander et al. 1993), whereas the template DNA methylation does not inhibit the Pol III transcription of a 5S rRNA gene (Besser et al. 1990). A recent study showed that loss of DNA methylation does not increase *Alu* RNAs in HeLa cells and B1 and B2 RNAs in mouse fibroblasts (Varshney et al. 2015), suggesting that their transcriptional regulation is independent of DNA methylation when a bulk of copies are considered together. Here, we analyzed the relationship between DNA methylation and expression at thousands of genomic B2 copies in cells showing high expression of SINEs (Fig. 4IJK). This revealed that, among B2 copies that have ≥3 CpG sites, hypomethylated copies were more preferentially transcribed. However, B2 copies with ≤2 CpG sites showed smaller expression difference by the level of DNA methylation. Evolutionarily young B2 copies have a higher number (typically, >5) of CpG sites, and our data suggest that transcription of these copies are likely regulated, at least partly, by DNA methylation. In contrast, genomic copies of the oldest B2_Mm2 subfamily contain less CpG sites, while providing more than half of B2 RNAs in spermatogonia (fig. 4*H*). Since no CpG site in B2 constitutes the sequence motifs (A- and B-boxes) of the Pol III promoter (fig. 1), DNA methylation-mediated transcriptional inhibition is likely due to recruiting methylated CpG-binding domain (MBD) proteins to the SINE regions (Varshney et al. 2015), rather than direct inhibition of Pol III binding. Given that CpG sites are hot spots of base substitutions during evolution (i.e.hypermutable), it is tempting to speculate that, young B2 copies are initially regulated by CpG methylation, and they gradually escape from this epigenetic regulation by losing the CpG sites during evolution.

During DNA methylation study of 51 genomic B2 copies, we found that the B2_25 copy resides at a boundary between the TSS surrounding hypomethylated region and the upstream hypermethylated region (fig. 5). We then took advantage of the absence of B2_25 in MSM, and showed that its removal results in disappearance of the boundary at the corresponding site, thus extending the hypomethylated region. Moreover, H3K9 acetylation level also differs across B2_25, and again its removal extends the acetylation domain (fig. 6). Consistent with such changes in DNA and histone modifications, the expression level of the neighboring gene, *Arcn1* in this case, was lower if B2 is present (fig. 6). The *Arcn1* gene encodes the delta subunit of the COP I complex involved in retrograde transport of proteins, such as endoplasmic HSP70 (i.e. BiP), from the Golgi apparatus to the endoplasmic reticulum (ER). Thus, it could be speculated that this activity is higher in MSM compared to B6 mice, which may allow for differentiation of the strains in terms of ER stress response. Another example of the B2-mediated boundary is found in the upstream region of thenicotinamide nucleotide transhydrogenase (*Nnt*) gene, where B2 insertion again downregulates the neighboring gene (fig. 7). The Nnt protein plays an important role in energy production and removal of reactive oxygen species in mitochondria (Hoek and Rydstrom 1988) and is implicated in diseases such as cancer (Albracht et al. 2011).

Genome-wide analysis of published ChIP-seq data revealed significant enrichment of B2 copies at boundaries of H3K9ac, H3K36me3, and H3K79me2 regardless of insertional polymorphism. Thus, it is conceivable that B2 copies carry a potential boundary activity, and consequently their retrotransposition can create new boundaries, some of which can be fixed later in the population. Based on our data on the allelic difference in the gene expression and chromatin states, it is speculated that in the ancestral state before the insertion of a B2 copy in a DNA hypomethylated and histone hyperacetylated promoter region, genes are expressed at a high level, and the expansion of the active chromatin becomes inhibited when a B2 copy invades this region. This scenario implies that B2 copies have reduced gene expression levels via retrotransposition into promoter regions during the course of rodent evolution. Note that this is not the phenomenon of gene silencing by heterochromatin formation, but rather it is reduction of expression by constraint of active chromatin. Whereas LTR retrotransposon insertions can induce heterochromatin formation and gene silencing (Rebollo et al. 2011), B2 insertions do not serve as a nucleation center for heterochromatin.

Regarding the proteins involved in the boundary formation at B2 copies, we found that the CTCF protein binds to the B2 copies at *Arcn1* and *Nnt*. Moreover, polymorphic and non-polymorphic B2 copies together constituted 16% of CTCF binding sites in ESCs; this finding extends from previous work indicating that B2-related SINEs generated CTCF (and ADNP) binding sites distributed within genomes (Bourque et al. 2008; Schmidt et al. 2012; Thybert et al. 2018; Kaaij et al. 2019), and suggests that B2-mediated proliferation of these binding sites is likely ongoing. However, in the case of the B2 copies at *Arcn1* and *Nnt*, we note that CTCF binding is not as strong as non-B2 CTCF sites. Moreover, the majority of B2 loci at boundaries of histone modifications did not bind to CTCF. Thus, CTCF-independent mechanisms of boundary formation also exist. TFIIIC is one of the basic complexes for Pol III transcription, binds to the B box in Pol III genes, and frequently resides within boundary regions (Noma et al. 2006; Raab and Kamakaka 2010; Yuen et al. 2017; Stutzman et al. 2020). In addition, because the consensus B2 sequences carry a TATA-box in the antisense orientation (fig. 1), it may be possible that bidirectional transcription by Pol III and Pol II promotes boundary formation, as suggested previously (Lunyak et al. 2007). We however note that B2 expression is very low in the tissue where we observed the B2-mediated boundaries.

While most SINE insertions are considered to be neutral (Lunter et al. 2006), some have exapted as *cis* regulatory elements (Bejerano et al. 2006; Sasaki et al. 2008; Franchini et al. 2011; Emera and Wagner 2012; Nakanishi et al. 2012; Nishihara et al. 2016). In all cases of such exaptation, SINE copies are divergent from the respective consensus sequences, indicating that accumulation of mutations are important for these SINEs to become functional. On the other hand, the sequences of *Arcn1*- and *Nnt-*neighboring B2 copies are almost identical to the consensus sequence of B2_Mm1a, with only a single nucleotide deletion and three nucleotide substitutions, respectively. In addition, many genomic B2 copies of young subfamilies provide binding sites for CTCF and/or ADNP. Therefore, B2 represents a case where the functionalization of a retrotransposon does not require subsequent mutations following retrotransposition. The activity of B2 to fine-tune gene expression by blocking the expansion of active chromatin provides evolutionary insights into the selective pressure for B2 retention in gene-rich genomic regions (Ichiyanagi 2013). Regarding B1 copies, which are also enriched in gene-rich regions, many were found at boundaries of H3K9ac, H3K36me3, and H3K79me2 (fig. 8). Whereas they did not bind to CTCF (fig. 9), some B1 copies have been shown to form a boundary by binding to the AHR and SNAI2 transcription factors (Roman et al. 2011). Therefore, it remains of interest to investigate whether Pol III, TFIIIC, or diverse TFs are involved in the functionalization of SINE families, which generates the epigenomic and transcriptomic diversity.

## Materials and Methods

### Identification of Polymorphic SINE Copies between the Two Strains

Sanger sequencing reads (typically 400–700 bp) obtained during the MSM genome sequencing (Takada et al. 2013) were compared to the B6 sequence by BLAST with options -gapopen 0 and -gapextend 1. Based on the sequence alignment, internal gap regions were extracted, where a sequence of 120 bp or longer was absent either in the reference or in the MSM sequence with its flanking regions being well aligned. Their sequences were analyzed by RepeatMasker (http://www.repeatmasker.org), and a SINE copy that occupied >80% of a gapped region were assigned as a polymorphic copy.

### Genome Sequence and Phylogenetic Analysis

Multiple alignment of 1,243 polymorphic B2 copies and 6,000 randomly selected genomic B2 copies (regardless of subfamily) was performed with Clustal Omega (Sievers et al. 2011). Using the alignment, a neighbor-joining (NJ) tree was constructed with MEGA5 (Tamura et al. 2011). The consensus sequence of B2_Mm1o was made from all sequences in the clade (fig. 2*B*, green), and the multiple alignment and NJ tree of the consensus sequences of the B2 subfamilies were generated with Clustal Omega and MEGA5, respectively. For re-annotation of genomic copies, the sequences of all B2 copies were obtained from the UCSC genome browser. RepeatMasker was run for these sequences using a custom library containing the consensus sequences of B2_Mm1a, B2_Mm1t, B2_Mm1o, and B2_Mm2. Statistics of nucleotide divergence from the respective consensus sequences were analyzed using the RepeatMasker outputs.

### Northern Blot Analysis

RNA samples were prepared from tissues using ISOGENE reagent (Nippon Gene), then ran on a 5% denaturing polyacrylamide gel, transferred onto a Hybond-XL membrane (GE Healthcare), and crosslinked by UV irradiation of 120 mJ. A radiolabeled probe was generated by 5'-end labeling of a synthetic oligonucleotide complementary to the 128–153 region of B2_Mm1a (5′-AGTACACTGTAGCTGTCTTCAGACAC-3′), where no nucleotide variation was found in the four consensus sequences. The membrane was incubated with the radiolabeled probe in Rapid-hyb Buffer (GE Healthcare) at 55°C for overnight. The membrane was then washed once with 2× SSC at 55°C for 10 min, then washed twice with 0.5× SSC at 55°C for 10 min. Radioactivity was detected on BAS2000 (Fuji Xerox). As an internal control, oligonucleotide probe (5′-CCTGCTCCGTTTCCGACCTG-3′) that hybridizes to 7SL RNA (191–210 of the 299 nt RNA) was used.

### Medium Length RNA-Seq (melRNA-Seq)

RNA from whole testes was prepared by using ISOGENE. Six hundred nanograms of RNA was either treated with 25 U of tobacco alkaline phosphatase (TAP) (Epicentre) for 1.5 h at 37°C or remained untreated for controls, and the reaction was stopped by phenol-chloroform extraction and ethanol precipitation. Another 600 ng of the total intact RNA was treated with 20 U of 5' polyphosphatase (Epicentre) for 30 min at 37°C, and the reaction was stopped by phenol-chloroform extraction and ethanol precipitation. TruSeq Small RNA Library Preparation Kit (Illumina) was then used for constructing sequence libraries; libraries were run on a 2% agarose gel and DNA fragments of 240–380 bp (corresponding to an insert size of 115–255 bp) were purified from the gel. After quantification, the libraries were sequenced on a MiSeq with MiSeq Reagent Kit v2 (Illumina) for 300 cycles using the 300 bp single-end mode, yielding about 3 million reads for each library (supplementary table S4, Supplementary Material online). The adaptor sequence was removed from the 3’ end of the reads by Cutadapt (Martin 2011), and reads that did not contain the adaptor sequence were discarded. The retained reads were used for RepeatMasker analysis. The results obtained from the TAP- and 5' polyphosphatase-treated libraries were very similar.

For preparation of the melRNA-seq libraries for spermatogonia, the cells were purified from the testes of B6 mice at postnatal day 7 by EpCAM labeling and cell sorting as described previously (Ichiyanagi et al. 2011). RNA was prepared by using ISOGENE, and 500 ng of RNA was treated with 25 U of RNA 5' Pyrophosphohydrolase (RppH, New England Biolab) for 1 hour at 37°C. The reaction was stopped by phenol–chloroform extraction and ethanol precipitation. Sequencing libraries were constructed by using NEBNex Small RNA Library Prep kit (New England Biolab), size-selected by gel electrophoresis, and sequenced on a MiSeq with MiSeq Reagent Kit v2 (Illumina) in the 300 bp single-end mode, yielding about 6 million reads for each library (supplementary table S4, Supplementary Material online). After removal of the adaptor sequence, these sequence reads were analyzed by RepeatMasker. Out of ∼11 million reads, about 250,000 reads were assigned as B2. The reads were then mapped to the reference genome using Hisat2 (Kim et al. 2019) to identify their genomic origins. About 100,000 reads were uniquely mapped (i.e. 40% mappability).

### DNA Methylation Analysis

Genomic DNA was prepared from spermatogonia, sperm, and somatic tissues using standard procedure, subjected to C-to-U bisulfite conversion, and subjected to touch-down PCR as previously described (Ichiyanagi et al. 2011). PCR products were cloned into pGEM-T easy vectors and at least 16 clones were sequenced for each locus.

### Chromatin Immunoprecipitation and Sequencing (ChIP-Seq)

Adult livers of B6, MSM, and F1 hybrid mice were minced into small pieces and fixed with 1% formaldehyde at room temperature for 15 min, and the fixation was stopped using glycine. Chromatin was prepared using the EpiSeeker ChIP Kit (Abcam) and incubated with anti-H3K9ac antibody (ab4441, Abcam). Chromatin was recovered using Dynabeads Protein G (Thermo Fisher Scientific), subjected to reverse crosslinking, and treated with proteinase K. ChIP-seq libraries were generated with the NEBNext Ultra DNA Library Kit (New England Biolabs) and sequenced on HiSeq2500 using the 50 bp paired-end mode. Approximately 40–90 and 22–70 million read pairs were yielded for ChIP samples and inputs, respectively (supplementary table S4, Supplementary Material online). Trim Galore (https://www.bioinformatics.babraham.ac.uk/projects/trim_galore/) was used for adaptor trimming, and Bowtie 2 (Langmead and Salzberg 2012) was used for mapping onto the reference genome (mm10) with the option “–maxins 10000” to efficiently map the MSM-derived reads around the indels. SNP data (Takada et al. 2013) was obtained from https://molossinus.brc.riken.jp/pub/MSM_2015.

### Gene Expression Analysis

RNA samples were prepared from tissues using ISOGENE (Nippon Gene), and cDNA was synthesized using PrimeScript RT (Takara Bio) and quantified by quantitative PCR with SYBR Premix (Takara Bio) using the Thermal Cycler Dice system (Takara Bio). For allelic comparison, cDNA was amplified by PCR and the products were sequenced using BigDye v3 (Thermo Fisher Scientific).

### Analysis of Published ChIP-Seq and WGBS Data

ChIP-seq peak positions for H3K9ac, H3K27ac, H3K4me1, H3K4me3, H3K36me3, H3K79me3, and H3K27me3 determined by the ENCODE project were downloaded from the UCSC genome browser (https://www.genome.ucsc.edu). Using RepeatMasker output downloaded from the UCSC genome browser, the genomic densities of SINEs, LINEs, and LTRs around the peak boundaries were calculated by a perl script.

ChIP-seq data for CTCF in liver (GSM4579731), for CTCF and ADNP in ES cells (GSE125129) (Kaaij et al. 2019), and for CTCF and CFCFL in round spermatids (GSM1817673 and GSM1817674) (Pugacheva et al. 2015) were obtained from Gene Expression Omnibus (GEO). After adaptor trimming by Trim Galore, the reads were mapped using Bowtie 2, and peaks were identified using MACS2 (narrowPeak) (Feng et al. 2012). Peak summits that resided within SINE copies were identified using BEDTools.

Whole-genome bisulfite shotgun sequencing data for spermatogonia (DRX020997) (Kubo et al. 2015) and ESCs (GSE41923) (Habibi et al. 2013) were downloaded from DDBJ sequence read archive (DRA) and GEO, respectively. After adaptor trimming by Trim Galore, the read mapping and calculation of methylation levels of individual CpG sites were carried out by bismark (Krueger and Andrews 2011).

These datasets are summarized according to the tissues and cells in supplementary fig. S6 (Supplementary Material online).

### Data Availability

All data generated for this paper has been deposited at NCBI GEO with the accession numbers, GSE156315 (melRNA-seq data) and GSE156316 (H3K9ac CHIP-seq data).

## Supplementary Material

Supplementary data are available at *Molecular Biology and Evolution* online.

## Supplementary Material

msab033_Supplementary_Data
