# Supplementary material for: B2 SINE Copies Serve as a Transposable Boundary of DNA Methylation and Histone Modifications in the Mouse
Source: Mol Biol Evol. 2021 Feb 16;38(6):2380–95. doi: 10.1093/molbev/msab033 (PMC8136502; doi:10.1093/molbev/msab033)
Supplement: msab033_Supplementary_Data [file msab033_supplementary_data.zip › Ichiyanagi_supplementary_figures.pdf]

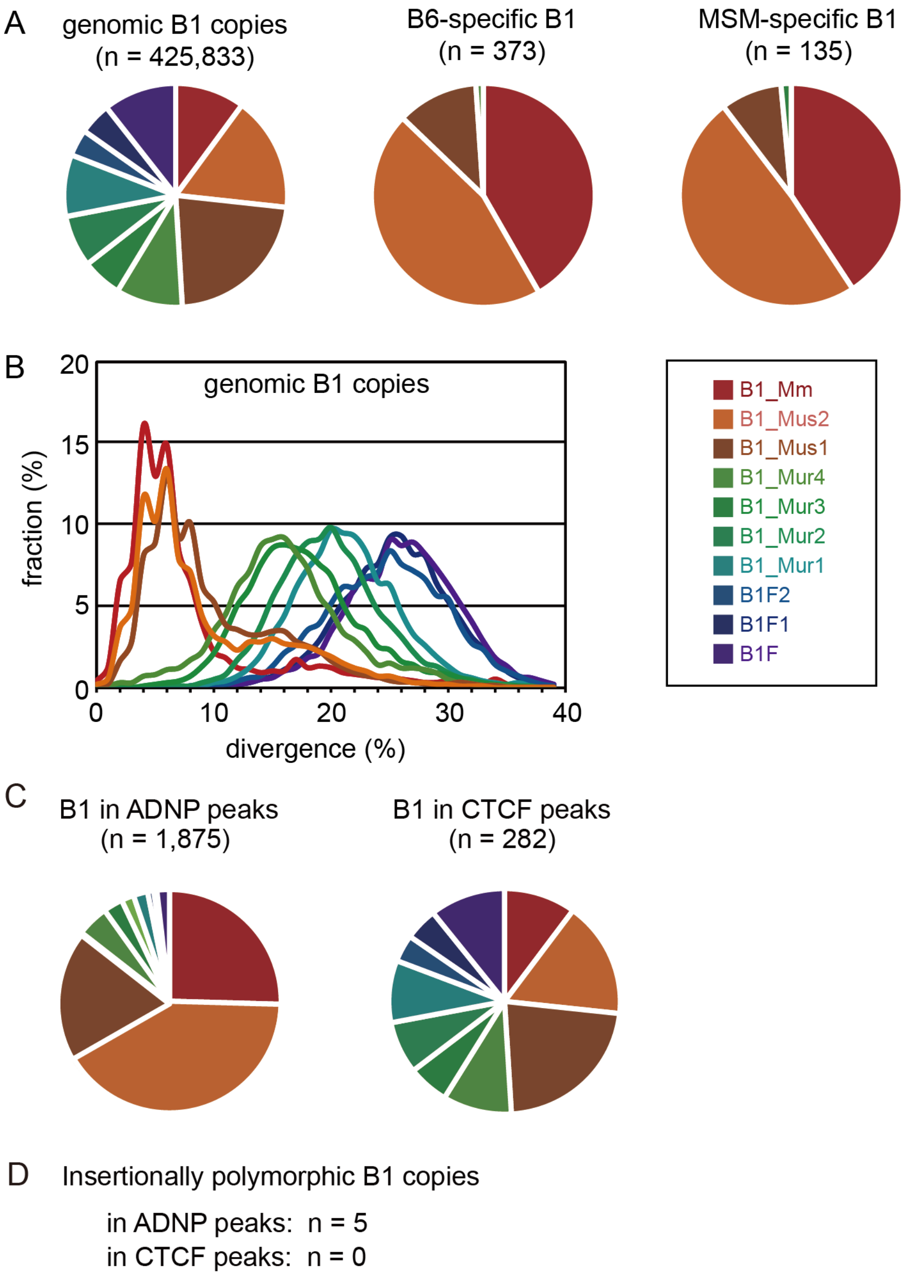

Figure S1. Statistics of genomic and polymorphic B1 copies.
A. Pie chart representations of the numbers for genomic (left), B6-specific (middle) and MSM-specific (right) B1 copies categorized by subfamily (see legend for color codes). **B.** Divergence of genomic B1 copies from the respective consensus sequences. **C**. Pie chart representations of the numbers for ADNP- and CTCF-bound B1 copies. **D**. The numbers of ADNP- and CTCF-bound polymorphic B1 copies.

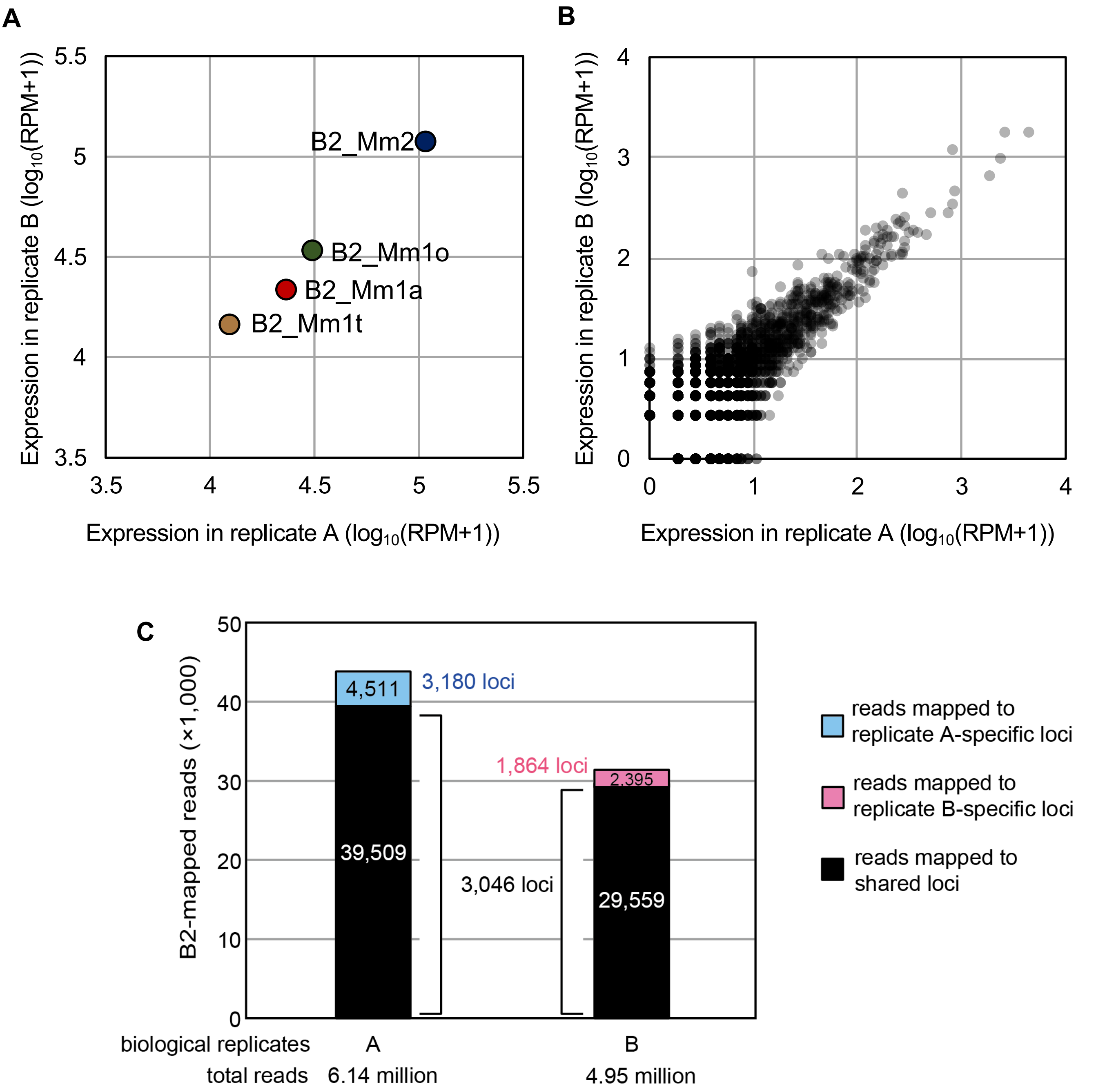

Figure S2. Reproducibility of meIRNA-seq.

A. Scatter plot of log10 expression levels of B2 subfamilies in the two biological replicates. **B.** Scatter plot of log10 expression levels of individual B2 loci in the replicates. **C.** meIRNA-seq data for spermatogonia were obtained from two replicates. The number of B2-mapped sequencing reads from shared loci are colored black. Those from replicate A-specific and B-specific loci are colored blue and pink, respectively. Most of the replicate-specific loci disclosed only one or two mapped reads, whereas mapped reads from highly expressed loci were detected in both libraries (see also panel B). Total number of MiSeq sequencing reads are shown on the bottom.

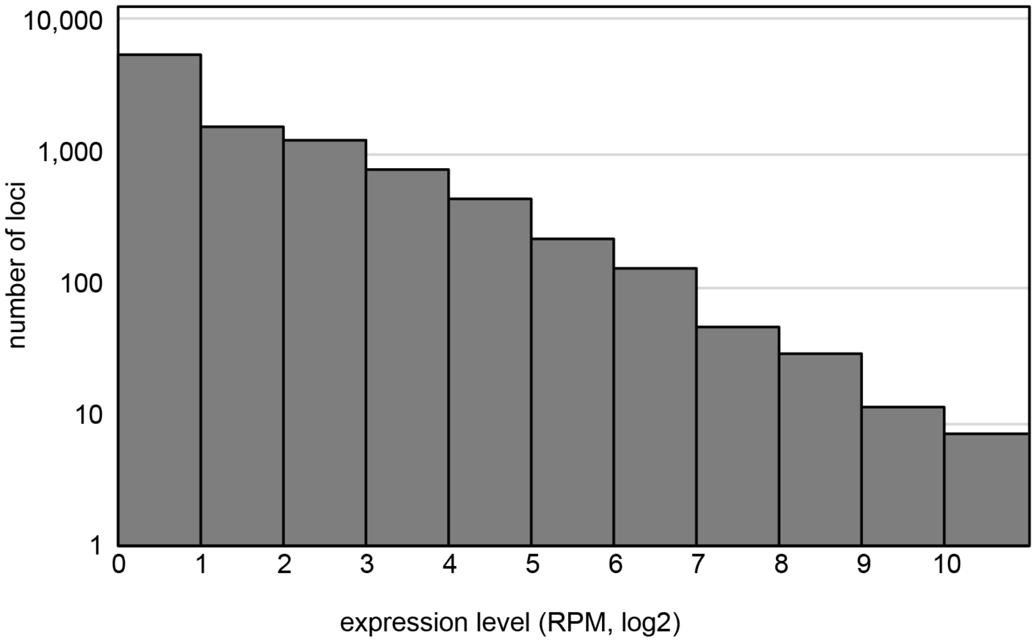

Figure S3. The Power-law pattern of expression for B2 loci.
The numbers of loci in log scale are shown for B2 loci categorized by RPM (reads per million 5.8S rRNA reads) in log scale. The RPMs of individual loci were calculated by using uniquely mapped reads only.

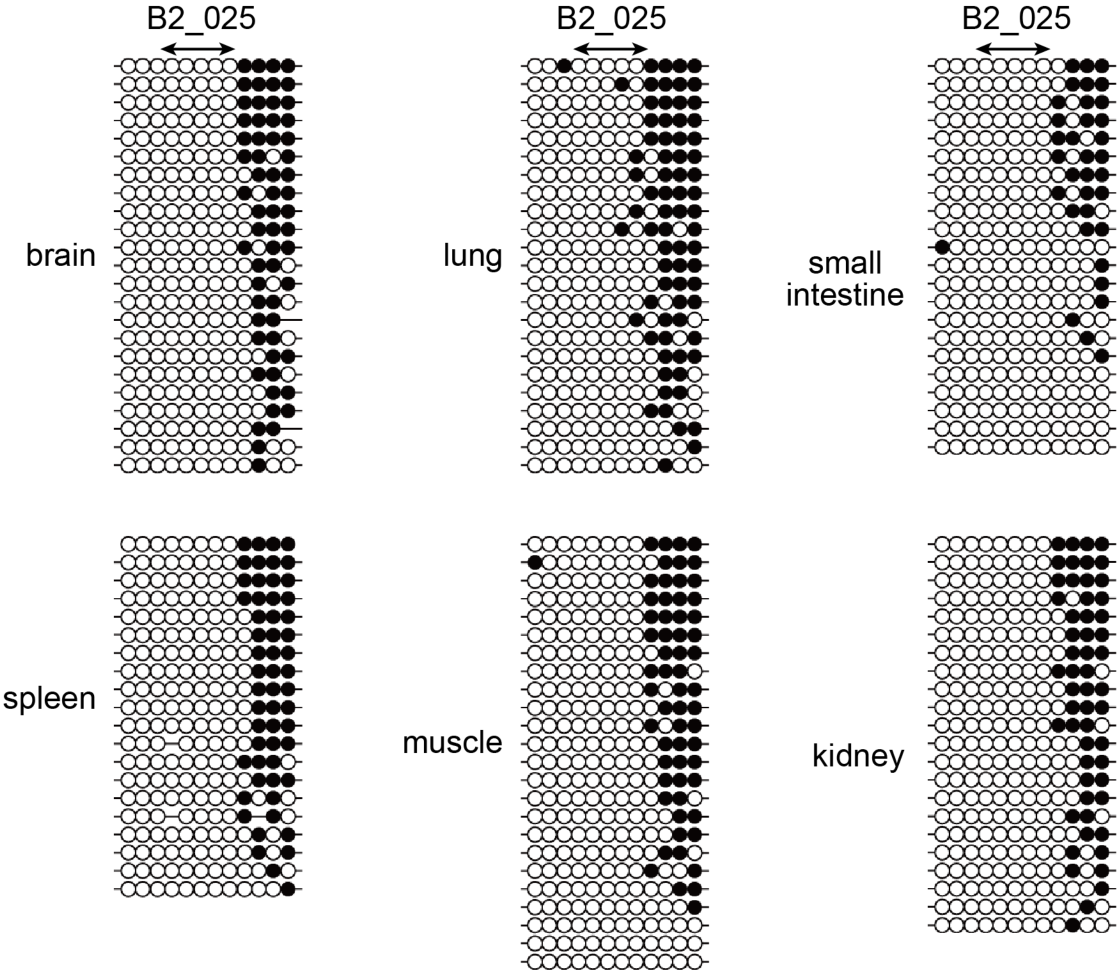

Figure S4. B2_25 is located at the DNA methylation boundary in a variety of tissues. Bisulfite-PCR sequencing data for tissues are shown. Open and closed circles represent unmethylated and methylated CpG sites, respectively. Each horizontal line represents a single PCR clone. The position of the B2_25 insertion is indicated by an arrow. Tissues are indicated on the left.

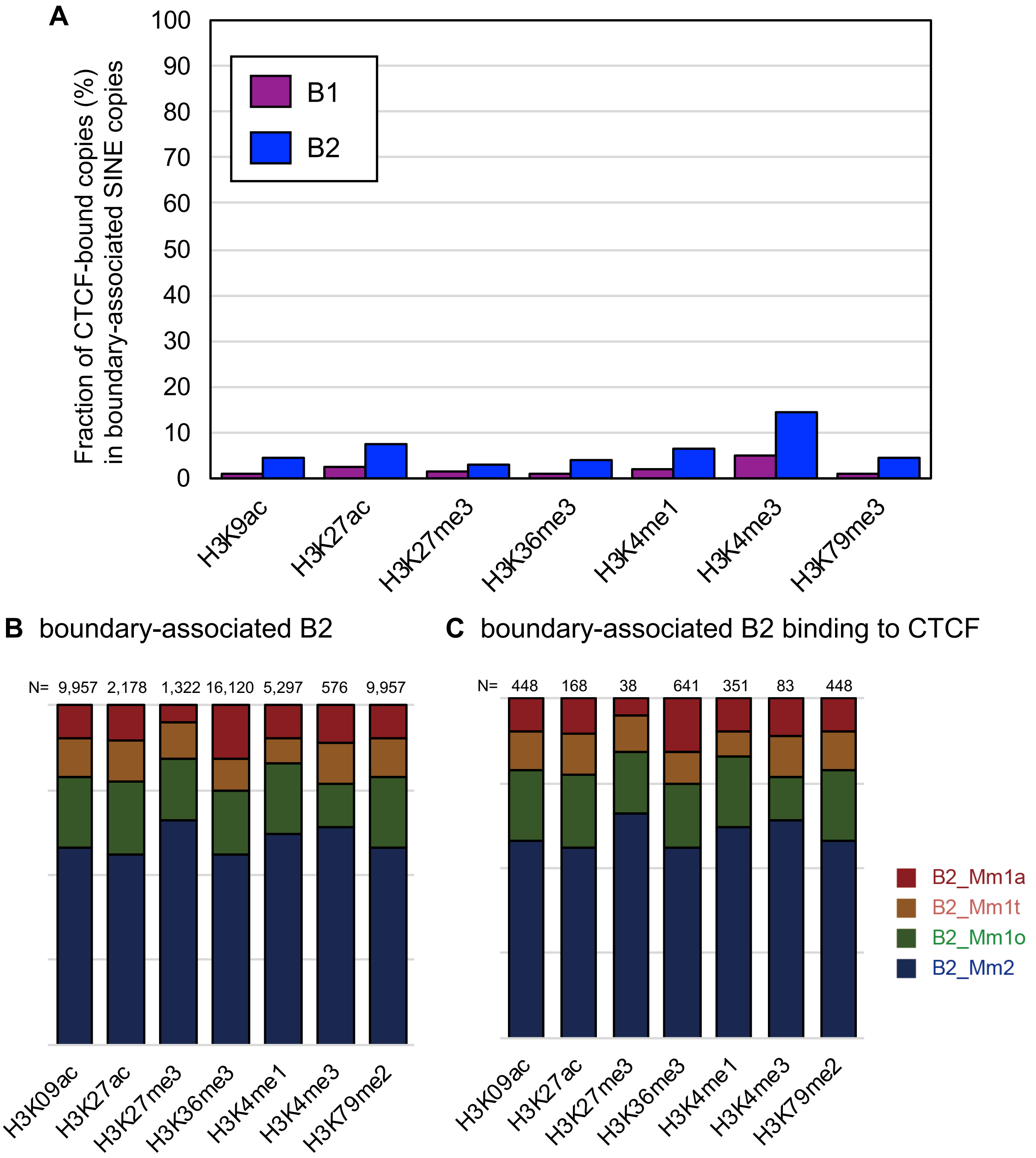

Figure S5. CTCF binding and boundary of histone modification at SINEs.
A. Fraction of CTCF-bound copies in boundary-associated SINE copies (which reside within 200 bp from a ChIP-seq peak end) are presented in purple and blue for B1 and B2, respectively. **B.** Fraction of each B2 subfamilies for loci associated with boundaries. **C.** Fraction of each B2 subfamilies for loci associated with boundaries and CTCF. The total number of B2 loci are shown on the top.

	liver (adult)	H3K9ac (this study, GSE156315)<br>various histone modifications (ENCODE)<br>CTCF (GSM4579731)<br>Locus-specific bisulfite PCR (this study)<br>RT-PCR (this study, *Arcn1* and *Nnt*)
germ cells	spermatogonia (postnatal day 7)	melRNA-seq (this study, GSE156316)<br>BS-seq (DRX020997)<br>Locus-specific bisulfite PCR (this study)
germ cells	spermatids (adult)	CTCF, CTCFL (GSM1817673, GSM1817674)
germ cells	spermatozoa (adult)	Locus-specific bisulfite PCR (this study)
	whole testis (postnatal day 7)	melRNA-seq (this study, GSE156316)
	whole testis (adult)	RT-PCR (this study, *Arcn1*)
	ES cells	CTCF, ADNP (GSE125129)<br>BS-seq (GSE41923)

Figure S6. Tissues and cells used for melRNA-seq, ChIP-seq, BS-seq, bisulfite-PCR, and RT-PCR analysis.
